# Alimentation in Health and Disease

**Published:** 1879-02

**Authors:** E. C. Angell


### Selections.

### ALIMENTATION IN HEALTH AND DISEASE.

By E. C. ANGELL, M.D.

"True temperance is true luxury."

Alimentation is a matter of primary importance in hygiene as well as therapeutics, and a correct system of dietetics is the corner-stone of human health; the judicious administration or withholding of food is often more than half the battle against disease. Under these truisms there lurks a formidable array of knotty problems which may be summed up in the interrogatory, What is correct alimentation? This, in the language of Lord Bacon, "is a matter that comes home to every business and bosom," or to speak with more anatomical strictness, to every abdomen. Not only must we determine what we shall individually eat and drink, but, as parents, many of us must settle the same question for the children whose welfare is one of our dearest interests, and, as physicians, the determination of the diet of the sick or convalescent is a constantly recurring duty.

This subject is treated more or less fully in every course of medical instruction. It has been the theme of abundant experiment and copious dissertation. Notwithstanding, the advocates of rival theories maintain, with equal ardor and sincerity, the most opposite views, and in actual practice ninety-nine out of a hundred are governed more by chance or custom than by any well conceived dietetic principles.

In ordinary health, men belonging to the medical profession, to other professions, or to no profession, are apt to indulge freely in whatever pleases their palates, with small regard to consequences; and even after disease has laid a heavy hand of warning upon them, the question is seldom what will be the most beneficial and strengthening, but rather can they eat this or that coveted luxury with impunity.

The average American does not give one-quarter of the attention to the welfare of his digestive system that he gives to the health of his dog or his horse. And what is the consequence? A nation of dyspeptics, and a greater mortality from the breaking down of the outraged digestive organs than even from the dreaded scourge of phthisis.

As a nation, we not only surpass all other nations in the multitude of our dyspeptics, but we have the poorest teeth and the best dentists. So long as the taste is pleased, the stomach may take care of itself, and the conviction is often forced upon us that far too many live to eat, and far too few eat to live.

A just appreciation of food esteems it not alone for the immediate gratification it affords, but rather for the health which it confers, the strength which it imparts, the life which it prolongs.

Far be it from me, however, to frown upon those who take pleasure in eating. The ascetic English nobleman who breakfasted on an emetic, dined on a cracker, and luxuriated once a week on an apple, is by no means a dietetic model to be recommended. I am in favor of an abundance as well as a variety, and firmly believe that the eating which is the most profitable for health and strength, as well as long life, is at the same time the most truly pleasurable.

I am quite willing to admit, however, that this doctrine is a little like that embraced in the familiar saying, "Be virtuous, and you will be happy." This is not disputed; but, as human nature is at present constituted, virtue is rather arduous, and the most of mankind prefer to take their chances without being over-righteous.

The fundamental question upon which the students of dietetics naturally divide, is whether man is naturally phytophagous or zoophagous, or both; whether he was intended to eat animals or plants, or to subsist upon either according to circumstances.

This is a question for the comparative anatomist, and admits of only one solution. It has recently, however, been admirably presented by Gustave Slickeysen, a German thinker, trained in the school of Darwin and Haeckel.

Slickeyson draws a minute comparison between the carnivora, the frugivora and the omnivera, represented by the tiger, the ape and the hog, and shows that man agrees in his physical characteristics far more closely with the tree-dwelling ape than with the blood-thirsty prowler of the jungle or the omniverous glutton of the sty.

In the arrangement and form of the salivary glands, in the conformation, number and structure of the teeth, in the size and shape of the stomach, in the length of the alimentary canal, and in many minor particulars, man shows a striking resemblance to the frugiverous gorilla, and an equally striking contrast to all of the carnivora, while the omniverous hog bears just such a mixed likeness to the carnivora on the one hand and the frugivora on the other, as might have been anticipated from the mixed nature of his food.

A careful consideration of these facts admits of little doubt that man was designed to dwell among trees and to live upon fruits, and curiously enough this conclusion of science tallies closely with the theological version of man's first estate.

Admitting these conclusions as correct, many will say it by no means follows that the diet which man in a state of nature was evidently designed to appropriate and assimilate, is not susceptible of great modification and improvement.

Man is certainly a being provided with extraordinary powers of adaptation, and doubtless soon discovered that by means of nets and traps, and the transforming influences of fire, the flesh of animals could be added to nature's *menu* of berries and fruits, leaves and seeds. Nor was there lacking, perhaps, good and sufficient reasons for this innovation, man being driven to this course by insufficient supplies of vegetable food, just as at the present day an animal diet is compulsory among the natives of the polar regions, or to cite an extreme case, shipwrecked mariners are sometimes forced to prey upon each other.

To choose an unnatural diet, however, upon compulsion, is very different from continuing that diet when improved cultivation of the earth has placed ample stores of the food nature designed for us at our command. The exiled trapper in the forest, with flesh, fish, and fowl of a dozen varieties on the one hand, and only a few handfuls of berries and nuts on the other, may very well group himself among the carnivora; but the more fortunate dwellers within the reach of farms, gardens, and orchards may profitably pause and inquire whether he shall nourish his body at second hand upon the carcasses of defunct animals which in their best estate are laden with effete elements, or at first hand upon the luxurious fruits, wholesome grains, and nutritious seeds in which Nature in her munificent wealth has stored up every element needful for his perfect nutrition.

It is not my purpose at this time to enter upon any of the moral or sentimental objections to the slaughtering of animals for human food, or to dwell at length upon the familiar argument against the use of flesh as an article of diet. From such undisguised evils as the dangerous trichinosis, the occasionally deadly poisoning of the unfortunate eaters of certain kinds of fish, to the disputed effects of beef tea in stimulating instead of strengthening convalescents, there is an array of damaging facts quite sufficient to convince the reason, though entirely inadequate to make headway against the appetite.

Appetites are artificial and hereditary, which is not strange when we consider the fact that each person has two parents, four grand-parents, and in each succeeding generation the number doubles from four to eight, then to sixteen, then to thirty-two, then to sixty-four, then to one hundred and twenty-eight, so that at the twentieth remove, omitting the factor of intermarriage, each person has over a million ancestors.

As appetites are transmitted like other hereditary tendencies, we soon come to regard supposed necessities, which arise from causes we are not apt to consider, as indispensible, which are in fact our worst enemies; and many who boast of being free men are really the most abject vassals.

The popular use of animal food, and the general opinion that it is strengthening, and that especially those who work hard cannot do without it, depends upon an acquired propensity as purely artificial and unnatural as the appetite for alcoholic beverages or the cultivated cravings for tobacco.

The average amount of beef-steak employed at a single breakfast produces an effect as essentially stimulating as a glass of wine; and those who think they cannot do without it are as much mistaken as that quite numerous class who think beer is necessary to their physical well-being.

This stimulation is curiously confirmed by the fact frequently noticed that upon changing from a diet largely composed of animal food to one containing essentially the same elements in a vegetable form, there is often a temporary feeling of discomfort and weakness, closely corresponding to that experienced by a reforming inebriate.

In choice, fresh, well-cooked beef from a healthy young bovine there is, aside from the effete elements it contains, a large proportion of the nutritious properties which in themselves are requisite to maintain the human body in its highest health and strength.

In the supply of carbon, for instance, it is nearly equal to a like weight of potatoes; and in supplying nitrogen, an ounce of such beef is more than equal to eleven ounces of potatoes.

These facts, casually considered, are calculated to give the impression that there can be no dietetic equivalent for flesh food, and that, whatever may be plainly urged against it, beef must remain the foundation and chief staple of our modern civilized dietary. This assumption, however, is erroneous.

The true basis of alimentation is not beef, but wheat; and in a natural and rational system of dietetics, wheat and the allied seed foods, including beans, lentils, peas, and rice, must take the place now usurped by the allied animal foods, including butter, cheese, eggs and milk.

Next to these should come the appetizing juicy fruits, and then the plant foods, which are neither seeds nor fruits, and which are generally styled vegetables. After these the various animal foods, and last of all the stimulating spices, beverages, and other food adjuncts, which unfortunately rank so high in our present ill-advised and ill-proportioned dietary.

The fowl or the joint occupies the post of honor, while the loaf is thrust to one side and too often forgotten, or overwhelmed in the crowd of seasoned dishes, or supplanted by cakes and pastries, from which the glorious vitality of the wheat has been successfully extracted. In fact, the miller and the baker between them have so contrived to emasculate the king of grains that we need not wonder that bread has been dethroned by beef, and even among the poor, by demoralized, ignoble pork and beer.

The true life giving, mental, moral and physical force-producing bread is neither more nor less than sound, ripe wheat when deprived of its thin outer silicious, innutritious husk, coarsely ground and mixed simply with water, and subjected to just that degree of kneading and baking that will suffice to prepare it for mastication, insalivation and the subsequent action of the gastric juice.

The lightness which is always sought in ordinary bread, and which is too often absent through the agency of yeast, through aeration with carbonic acid gas, saleratus, or baking-powders, is more effectually secured by kneading the dough into small rolls a little larger than the largest maccaroni.

These rolls have received the appellation of sticks, which is not inappropriate, as they will be found upon proper employment to "stick to the ribs" in a manner paramount to any other alimentary substance. There is additional fitness in denominating these rolls as sticks, as they may be said emphatically to constitute the staff of life. The same amount of money ordinarily paid for an average loaf of bread, which is comparatively worthless, will purchase as much attrition flour as will, with the requisite manipulation, produce bread enough to supply six times the strength.

These crisp sticks are characteristic as the Britons' plumb pudding, the Italians' maccaroni, or the Russians' pumper-nickel, and far superior to either as a suitable food for all ages, classes and conditions, and every way worthy to become a distinctive national alimentary staple.

The value of this bread cannot be easily exaggerated, and its habitual employment in a family I would consider a greater gain than the abandonment of animal food in favor of ordinary white bread, cakes and pastries.

There are serious objections to bakers' bread from the fact that the flour from which it is made contains little except starch, and is still further deteriorated by fermentation. It is always surcharged with water, commonly mixed with an excess of salt, and often adulterated with alum, ammonia, borax, potatoes, soda, soap and other objectionable substances.

The average household bread, though less adulterated, has little value, is rarely masticated, and even when buttered is swallowed with so much difficulty that it is generally washed down with hot drinks. It is a fruitful source of consitpation, which succeeds to diarrhœa, and ultimately to dyspepsia and its numerous attendant disabilities.

In the sticks we have every nutritious element of the grain, with no fermentation, with no cryptogamic vegetation; and we are equally certain that no deleterious chemical or mineral ingredient has been added. We have, furthermore, a substance that must be chewed, as it cannot be swallowed without thus insuring due mastication, insalivation, and correspondingly increased ease of digestion.

Attrition, or cold-blast wheat, coarsely ground and unbolted, contains all the natural nutritious elements of the wheat. And aside from this advantage, it possesses the mechanical properties that distend the intestines and thereby affords the requisite stimulus to peristaltic action, so that this bread, employed at proper intervals, in proper quantity, and when properly masticated, promotes digestion, and may be considered in no small degree antidotal to dyspepsia and its attendant consequences. For children it is especially valuable, and its substitution for common bread, and the use of fruits instead of flesh food, until the deciduous teeth shall have given place to the permanent dentures, would be of incalculable benefit and would contribute to supply a better quality of teeth. The early loss of these organs furnishes conclusive evidence, even without other proof, that the prevailing system of dietetics is radically wrong.

Cracked wheat, corn, oat and rye meal and other mushes have long been the main dependence of those who have believed in dispensing with flesh; but they are not to be compared with the bread I have described, except for those who are too young or too old for teeth. The chief objection to mushes is that they are generally gulped without chewing.

After placing one trencher of wheaten sticks in its proper position as the central attraction of the table, the next step is to fortify it on every hand with an array of fruits so attractive and varied as to prevent desertion back to other less nutritious substances.

The variety of food of this character which may be easily obtained is calculated to surprise any one who has not given the subject attention, as there are few places in this country in which a family sufficiently prosperous to have a daily supply of chops, steaks or joints may not replace them with an equal variety of fruits.

An important incidental advantage of this diet arises from supplying in the form of fruit-juices nearly all the liquid the system requires, and this too with water as pure as that obtained through the most careful distillation, and thus obviates the temptation to the common mistake of drinking at meals. The delicate and delicious flavors of fruits appetize and afford a natural zest, and check the disposition to indulge in condiments; and, moreover, despite the large proportion of water contained in fruits, there is in most of them no inconsiderable amount of nutrition.

Apples have in all times occupied a prominent place as food, not only because of their intrinsic merits, but from their historical relation to the garden of Eden. This excellent fruit has alone in many instances for months, and in not a few for even years, sustained life comfortably and completely in full vigor. This fruit is generally abundant and cheap, and in the many kinds furnished by improved cultivation far surpasses any other, affording upwards of two thousand varieties. It ripens early and late, and may with comparative ease be preserved in good condition during the greater part of the year. The plump limbs and rosy cheeks of children fed freely upon apples would be a revelation to those who have not investigated the advantages of fruit as food.

Neither apples nor other fruits, however, should be eaten by adults or children except at meals. There is no objection to baking, steaming, stewing or roasting the apples, although the best results may be expected from the fruit uncooked.

Pears are less abundant and more perishable, and in less variety, and are correspondingly more costly. They hold a place, however, of too much importance to be overlooked. This fruit cannot be too highly esteemed for its distinctive flavors, and it has been claimed by some writers that pears have a specific value in consequence of their containing no inconsiderable amount of iron.

Peaches are generally abundant and cheap for several weeks, and those who have once used this substantial food will not fail to provide it in unlimited quantities. All persons of gouty diathesis should make this fruit their chief alimentary staple during its season; and from the relatively small amount of sugar it contains, it is the most available of all fruits in diabetic diseases. I have never yet met so confirmed a votary of animal food that he could not be tempted by wheaten sticks and the delicious Crawford Superb, or the appetizing Jersey Rareripe.

Those unaccustomed to fruit as food might be more or less apprehensive of discomfort and disturbance from its use in hot weather, but I have rarely failed to find it a sovereign corrective to functional disorders of the digestive system when taken at proper intervals as a part of, or as constituting, the entire meal.

Fruit eaten between meals or at dessert, when too much dinner has been eaten already, is quite another matter, and a digestive system that would not rebel against such abuse must be sadly deteriorated.

Another fruit of generally recognized excellence is the grape. This is not only a highly esteemed table favorite, but its good qualities have given rise to grape cures.

Cherries, currants, plums and prunes, though less abundant, are nevertheless worthy of attention in their season, and when carefully dried are available for all seasons.

The strawberry needs no enlogium, for everybody is in accord with quaint old Fuller, that "doubtless God might have made a better berry, but doubtless God never did."

Blackberries, raspberries and whortleberries, that follow, though not such universal favorites, are scarcely inferior in excellence, the blackberry especially being among the most salutary of fruits.

Figs are among the most highly esteemed of American fruits. They are abundant throughout the Southern States, but do not admit of transportation, and when dried are inferior to those of Italy and Turkey.

The orange is well known to be one of the most agreeable of common fruits, especially grateful and refreshing, and generally admissible under almost every condition of sickness, convalescence or health.

Bananas, the universal food of the tropics, and the daily bread of whole tribes of men in its native clime, is one of the most substantial of fruits, the "fig" of Paradise, named in honor of its having been the chief food of the wise men of the East, the Brahmins. It consists largely of starch, and, in addition, of a considerable amount of nitrogen, and is therefore of superior food value to the ordinary starchy preparations of arrowroot and sago, which require cooking.

Dates in their native home hold the same relation to the multitude as the banana—they are the bread of the desert, and cannot be outranked in nutrition and wholesome qualities among dried fruit. Without considering fruit in further detail, and conceding, for the sake of the argument, that we have established as a central attraction pure wheaten, unadulterated bread, and around it a generous supply of fruit, it only remains to fill the outskirts of the table with simple preparations of barley, corn, oats, rice and rye, and especially of beans, lentils and peas, which are rich in nutritious elements not largely supplied in either grains or fruits. According to Josephus, lentils constituted the food that sustained the builders who constructed the Pyramids.

Careful consideration will discover that our table, to use the current banquet phrase, is groaning beneath its burden of the highest products of the vegetable kingdom; and this is the fountain head, the source of all nutrition.

Here is nothing of a mineral nature, not even common salt, except as Nature has distributed it; here is nothing from the animal world, not even butter, eggs or milk. In fact, here is nothing but fruits and seeds, the various grains ranking as seeds; and in these, I repeat, we have the very highest products of the vegetable kingdom, comprising in themselves, in the best and purest form, every requisite element for the complete and correct alimentation of man. I look upon this regimen of seeds and fruits as the ideal diet, to be kept always in view, and approached in practice as closely as circumstances will admit.

Those who use this regimen will have little desire and less occasion for alcohol, opium and tobacco; and all who have become the unwilling slaves of either of these agents may most easily and most readily effect their emancipation by adopting the ideal diet I have described.

Outside of this range of food we have the potato, which is almost a seed; and next perhaps in rank, the tomato, which is almost a fruit. Beets, canteloupes, carrots, cauliflowers, melons, squashes, turnips, parsnips, pumpkins and other vegetables might be added for the sake of variety. Any garden products, however, which depend upon sauces and seasoning to make them palatable, are not worth the cooking, and should only be used from necessity.

The extensive use of brandy and wine sauces, with gravies and rich seasonings, are practically so many confessions on the part of flesh eaters of the truly insipid, if not offensive, flavors of their boasted viands. Many varieties of game are much to be preferred, when fresh, to our domestic animals; and young meats, like lamb and veal, are highly objectionable; and notwithstanding Charles Lamb's rhapsodies on roast pig, I would rigidly enforce the Mosaic law against the obese swine. I fully concur with the sentiment expressed by Dr. Adam Clark, who said, "If I were to make an offering to his Satanic majesty, it would be a hog stuffed with tobacco."

Fish, notwithstanding its extensive use, has little value as food, especially when long out of its native element, and when salted, is probably, next to pork, the most damaging of animal aliments, and should be used only as a matter of positive necessity.

Milk, cream, butter, cheese and eggs are forms of animal food which many opponents of flesh, fish and fowl have freely used. Milk, necessary as it is when naturally secreted for the infant, is not always acceptable to the adult. In fact in many instances it taxes the digestive system to an extent that amounts to positive injury. The presence of the permanent teeth may be regarded as evidencing the importance of the use of such food as will furnish enjoyment for these very important organs, for want of which, like the unused key, they become injured and impaired.

Cream is not only the most universally grateful, but is unquestionably the most salutary of animal fats, and when fresh and newly transformed into butter—before the addition of salt—while less salutary, is to many made still more acceptable. The general burlesque upon this substance which is found in the average boarding-house, and is heavily salted the better to disguise its significant strength and to increase its weight, should be banished from civilization.

Cheese is certainly clogging, and I think is rarely relished by those who have been fortunate enough to retain normal tastes, and the acme of perversion may be said to be reached in the consumption of limburger.

Eggs are too highly concentrated for general use, the albumen, as commonly cooked, taxing the digestive system so as largely to detract from their advantages.

So far as the various beverages are concerned that usually find places on our tables, it may be already inferred that I would banish them all, water included, and especially iced water, in favor of juicy fruits, and with the free employment of fruits and vegetables none will be needed.

Water, being the universal solvent and the vehicle of all nutrition, some two hours after eating, may be drank as freely as thirst requires. As much of this fluid finds its way into the blood, the purer it is the better.

Mineral waters, whether artificial or natural, should be avoided under all circumstances, unless pure water cannot be procured. Pure water is always grateful to pure tastes when needed, and it is only to perverted tastes that it seems flat and insipid. Artificial or natural mineral waters generally contain common salt in such amount that they fail to satisfy thirst, and most of them are impregnated with other salts that are damaging, deleterious and destructive. Arsenic figures in some of them quite conspicuously.

Coffee and tea are much more injurious than are generally supposed. Strong coffee will at least promote, if not indeed cause gout, and its continued employment seriously impairs the blood, irritates the kidneys, and deranges the nervous system. Tea, with its abundant adulteration, is more harmful. Chocolate, habitually used, is probably more damaging than either. For those who cannot be made to believe that it is profitable or possible for them to do without hot drinks, I believe in some instances induced them to employ parched wheat as the least injurious of unnecessary beverages.

Alcoholic potations and preparations in all their forms, I have no doubt, aside from their stimulating effects, influence the vital process of retarding the metamorphosis of tissue, thus retaining in the system effete principles which it would be much better to be well rid of.

The use of condiments, sauces and spices is a confession of poor cooking, worn-out appetites, and jaded tastes, which awaken commiseration in those who know the value and sweetness of wheat, and can truly relish the flavor of a peach, a pear, or a pine-apple.

Among the most mischievous and universal of seasonings is sugar, which is sufficiently abundant in fruits, grains and vegetables. It is hard to estimate the amount of discomfort and disease which arises from this one source, aside from the cost, which, though large, is the smallest of the detrimental consequences, even when free from positively poisonous adulterations.

The damaging effects of this agent are admirably enunciated by Victor Hugo when he addresses woman in the following language: "Remember, you eat too much sugar. You have but one fault, oh woman! It is that of nibbling sugar. Oh, consuming sex! [Now listen attentively. Sugar is a salt. Every salt is dessicating. Sugar is the most dessicating of all salts. It sucks up the liquid from the blood through the veins. Thence comes the coagulation, thence the solidification of the blood, thence tubercles to the lungs, thence death. This is why diabetis borders on consumption. Crunch no more sugar, therefore, and you shall live!"

The next in importance and next in mischief is common salt, and notwithstanding the weight of popular and scientific opinion on the subject, I think we are far better off without it, other than as distributed by Nature in fruits, grains, and vegetables. It may not debauch the taste to the same extent as its associates, pepper and mustard, but conjoined, they constitute a trinity which cries loudly for drink, for more drink, and ultimately for strong drink, and it is hard to estimate how much these allied enemies of our race have to do in making the drunkards who people our inebriate asylums, as well as our criminal courts and prisons.

Instead of the vinegar bottle, lemons should be employed, and the whole array of pickles is little more than a series of insults to the stomach, which it is very slow to digest. Catsup and hot bottle sauces are equally objectionable, and are simply ingenious inventions to hide the otherwise offensive flavors of unpalatable and unwholesome animal aliment.

Aside from the nature of the food there are other points scarcely less important in alimentation. Quality as well as quantity deserves consideration, as well as self-denial. A considerable objection to animal food arises from its concentrated form, and the corresponding case with which too much is eaten before any sense of fullness gives warning to stop, and the artificial fillip given to the appetite by sauces and pickles double the temptation.

The fruit and seed foods I have pointed out as constituting the ideal diet may be used abundantly with much less risk of surcharging the stomach. This advantage the votaries of animal food must forego while they make up their minds to pay for their pleasure by enduring with increased frequency the pangs and penalties of an outraged and demoralized physical organization.

Too often, as well as too much, is a common error in dietetics. Two meals a day instead of three is a golden rule in alimentation. Breakfast at nine and dinner at five is a dietetic time-table that cannot easibly be improved for business or professional men. Lunch between is a habit rather than a necessity, and by sedulously avoiding all food at other times, by which I mean to include fruits, and giving the digestive system at the hours named a fair square task, with an intervening period of absolute rest, the best possible condition of the system may be maintained, and by this means alone can we confidently look for a sound mind in a sound body, for a poor stomach necessarily produces a poor brain.

If meats are to be employed at all, they should be restricted to once a day, and then in small amounts, choice in condition and quality, and of a single variety, and used rather as a relish than, as too often, as the main feature of the meal.

